# The effects of clinical and sociodemographic factors on survival, resource use and lead times in patients with high-grade gliomas: a population-based register study

**DOI:** 10.1007/s11060-018-2899-0

**Published:** 2018-05-30

**Authors:** Jenny Bergqvist, Hanna Iderberg, Johan Mesterton, Roger Henriksson

**Affiliations:** 1 Institution of Oncology-Pathology, Karolinska Institutet, Stockholm, Sweden; 2 Capio St Görans Hospital, St Görans plan 1, 112 81 Stockholm, Sweden; 3 Ivbar Institute AB, Hantverkargatan 8, Stockholm, Sweden; 4 Medical Management Centre, Karolinska Institutet, Stockholm, Sweden; 5 Regional Cancer Centre Stockholm Gotland, Stockholm County Council, Stockholm, Sweden; 6 Department of Radiation Sciences and Oncology, University of Umeå, Umeå, Sweden

**Keywords:** Glioma, Brain neoplasm, Comorbidity, Registries, Health resources, Sociodemographic factors

## Abstract

**Background:**

Previous studies indicate an effect of sociodemographic factors on risk for being diagnosed with, as well as on survival of cancer in general. Our primary aim was to analyse sociodemographic factors, resource use and lead times in health care after diagnosis with high grade malignant glioma (HGG) in a large population based cohort.

**Methods:**

A register-based study using several unique high-coverage registries. All patients over the age of 18 diagnosed with HGG in the Swedish Stockholm–Gotland region between 2001 and 2013 (n = 1149) were included.

**Results:**

In multivariable cox proportional hazard model of survival, older age, male sex and high tumour grade were associated with worse survival. No significant differences could be seen related to country of birth. A high disposable income was associated with better survival and fewer occasions of pre-diagnostic inpatient care. Older age and comorbidities were correlated with a significantly increased number of outpatient visits the year before HGG diagnosis. In addition, male sex, being born outside Sweden was associated to a higher number of outpatient visits the year after diagnosis in multivariable analysis. Leadtime from diagnosis (first suspicion on brain scan) to surgery showed that the oldest patients, patients with comorbidity and patients born outside Europe had to wait longer for surgery.

**Conclusions:**

Sociodemographic factors like education, income and country of birth have impact on care processes both before and after the diagnosis HGG. This needs to be acknowledged in addition to important clinical factors like age, comorbidity and tumour grade, in order to accomplish more equal cancer care.

## Introduction

Diagnosis and treatment of high grade glioma (HGG) have been improved during the last years, still the prognosis is poor. In fact, the 5-year survival for the around 400 patients annually diagnosed with HGG in Sweden (total population 10 million), is lower than 10% [1]. Treatment is based on patient and disease specific prognostic factors like age, performance status, histologic grade and tumour molecular profile. Characteristics of the tumour/disease, health status of the patient as well as treatment regimen clearly affects the outcome [2, 3]. Previous publications suggest that sociodemographic factors are associated with how individuals respond to, acknowledge symptoms and thereby also affect time to diagnosis [4]. In addition, an increasing number of reports present that comorbidities and sociodemographic factors such as education, income level or country of birth, not only affects the risk of getting a disease [5, 6] but may also have an impact on survival [7–11].

The risk of treatment bias based on old age for patients with glioblastoma as well as for other types of cancer is well known, since older cancer patients tend to be offered less aggressive treatments. However, omitting radiotherapy in the treatment of glioblastoma seem to be associated with, not only older age, but also with race, unmarried status and lower annual income [12]. However, the studies of HGG in relation to sociodemographic factors are relatively few, often based on relatively small patient cohorts and rarely include resource use. In addition, they show some contradictory results.

Therefore, this study was performed on a large high quality unique database combining data from multiple registries with detailed information (e.g. all diagnosis set and procedures performed at hospitals, cause and date of death, sociodemographic information and patient and tumour characteristics) on all citizens.

Our primary aim was to analyse sociodemographic factors in relation to survival, use of health care resources and lead times in the care process for Swedish patients with HGG.

## Methods

### Study population and data sources

This register-based study included data from national and regional databases with time of recruitment based on the time period 2001 to 2013. However, throughout 2015 was used to follow patients in terms of resource use and survival after data of diagnosis.The study population was all patients in the Stockholm region, diagnosed with high-grade glioma according to SNOMED histopathological classification [13] reported in the Swedish Cancer Register (SCR). The SCR keeps record of all newly detected tumours in Sweden and has a coverage rate above 95% for malignant tumours of which 99% are histologically confirmed [14]. All patients diagnosed between 2001 and 2013 and identified in the SCR [15] were included. The Stockholm region includes around 23% of the Swedish population (2.3 million citizens 2015) [16].

Through record-linkage using the patients’ personal identification number, data were extracted from: The Swedish Cancer Register, the National Cause of Death Registry (information on date of death) [17], Patient administrative systems (PAS; information on all healthcare visits and procedures in inpatient and outpatient care) [18], the Swedish Brain Tumour register (SBTR) (cancer treatment in detail and lead times) [1] and Statistics Sweden’s population data (educational level, disposable income and country of birth) [16]. The classification of HGG is based on the WHO criteria from 2007 [19]. The regional Ethical Review Board in Stockholm approved the study protocol (Dnr 2012/1236-31/4).

### Study variables

#### Health outcomes

Survival analysis was performed by calculating the number of days from diagnosis until date of death or date of loss-to-follow up based on information from the National Cause of Death Register.

#### Resource use

Information regarding diagnoses and procedures in inpatient and outpatient care, only available for patients living in Stockholm (n = 845), was extracted from PAS for the Stockholm region. Inpatient days and outpatient visits 1 year before and 1 year after the date of diagnosis were calculated. Inpatient care are days spent in hospital during hospital admissions. Outpatient care are visits to an outpatient specialist clinic (often to a doctor, but can include visits to other professions like nurse etc.), but not to the primary care or general practitioner. Only patients living in Stockholm at the time of diagnosis were included in analyses of resource use.

#### Care process: lead times

Analysis of lead times (in days) between date of diagnosis to date of surgery as well as between date of surgery to start of non-surgical cancer treatment (radiotherapy, chemotherapy or other non-surgical cancer treatments), all reported in SBTR.

#### Patient characteristics

A set of clinical and sociodemographic variables was defined (age, year of diagnosis, sex, comorbidity, educational level, income level, country of birth and tumour grade) as relevant when studying the effect of clinical and sociodemographic factors on outcome and resource use. Four pre-defined time periods were used when stratifying patients by year of diagnosis (2001–2004, 2005–2007, 2008–2010 and 2011–2013). Data on age at diagnosis (defined in four categories: 18–39, 40–59, 60–69, 70–) and sex were taken from SCR. The category high-grade glioma includes both grade III (e.g. anaplastic astrocytoma) and grade IV gliomas (e.g. glioblastoma multiforme), which are known to have different prognosis. For comorbidity analyses the Elixhauser comorbidity index was used [20], which consists of a predefined set of 31 comorbidity categories and data was extracted from PAS. Patients were classified as comorbid if at least one comorbidity diagnosis was registered in the PAS (inpatient, outpatient or primary care) during two years before cancer diagnosis. Information of educational level (categorized as elementary, high school diploma or university degree), disposable income (adjusted for family constellation and categorized as low, medium or high income) and country of birth (categorized as born in Sweden, born in Europe (not Sweden) or born outside Europe) was obtained from Statistics Sweden.

#### Statistical analysis

Unadjusted survival over time was estimated using Kaplan–Meier analysis and stratified by age, comorbidity, sex, tumour grade, educational level and disposable income level. Tests of statistical significance of differences between groups were performed using log-rank test for equality of survivor functions. The univariable and multivariable effect of a number of selected clinical and sociodemographic variables (age, year of diagnosis, sex, comorbidity, educational level, income level, country of birth and tumour grade) on survival, resource use and lead times was evaluated. A Cox proportional hazards regression model was used to calculate adjusted hazard ratios (HRs) and 95% CIs for the univariable as well as multivariable effect on all-cause mortality. A negative binomial regression model was used to estimate the effect (univariable and multivariable) of the same set of case mix variables on resource use before and after high-grade glioma diagnosis as well as on a set of important lead times (days from diagnosis to surgery, days from surgery to histopathological report and days from surgery to start of non-surgical cancer treatment). Incidence rate ratios (IRR) and 95% confidence intervals as well as p-values for each case mix factor are reported. IRR should be interpreted as the relative difference of days or outpatient visits when the explaining factor is changed by one unit. Statistical analysis was carried out using STATA 13.1 (Stata Corporation, College Station, TX). To be included in analyses of resource use after diagnosis, the patients have to be alive after 365 days, (n = 454).

## Results

### Patient characteristics

There were 1149 patients diagnosed with HGG in the Stockholm Gotland Region during 2001–2013 out of which 845 were living in Stockholm at the time of diagnosis. During the observation period, 1005 patients died. Total time from diagnosis until death or end of follow-up was 3044 person-years, with a median follow-up time of 457 days. The total study population and the Stockholm subpopulation had similar characteristics. Average age at diagnosis was approximately 57 years, range of 19–92 years. The majority of patients, approximately 60% were men. In total, 375 (44%) of the patients suffered from at least one comorbidity based on the Elixhauser comorbidity index definition. Hypertension was the most common comorbidity followed by neurological symptoms/diseases (ataxia, degenerative diseases, Parkinson’s disease, MS, epilepsy, tremor), depression, other tumours and diabetes (data not shown). Over 80% (687) were born in Sweden and only 6% (49) were born outside Europe. A university degree was registered for 328 (44%) of the patients. Further details of the study population in total and the subpopulation in Stockholm are summarised in Table 1.

**Table 1:** Descriptive statistics of study population in total n = 1149 and the Stockholm population n = 845 with data on health care resource use

Variable	Category	No. patients Stockholm	%	No. patients total	%
Number of patients		845		1149	
Age (average)		57.6		56.6	
Age category	18–39	90 (845)	10.7	141 (1149)	12.3
	40–59	332 (845)	39.3	456 (1149)	39.7
	60–69	284 (845)	33.6	379 (1149)	33.0
	70–	139 (845)	16.5	173 (1149)	15.1
Year of diagnosis	01/04	223 (845)	26.4	268 (1149)	23.3
	05/07	171 (845)	20.2	222 (1149)	19.3
	08/10	224 (845)	26.5	337 (1149)	29.3
	11/13	227(845)	26.9	322 (1149)	28.0
Sex (male)		513 (845)	60.7	684 (1149)	59.5
Comorbidity (%)**		375 (845)	44.4		
Education level	Elementary	153 (818)	18.7	210 (1118)	18.8
	High school diploma	337 (818)	41.2	472 (1118)	42.2
	University degree	328 (818)	40.1	445 (1118)	39.8
Disposable income	Low	306 (818)	37.4	430 (1118)	38.5
	Intermediate	337 (818)	41.2	478 (1118)	42.8
	High	175 (818)	21.4	220 (1118)	19.7
Country of birth	Born in Sweden	687 (841)	81.75	977 (1149)	85.0
	Born in Europe (not Sweden)	105 (841)	12.5	125 (1149)	10.9
	Born outside Europe	49 (841)	5.8	52 (1149)	4.5
Grade IV		725 (845)	85.8	968 (1149)	84.3

All the data included are extracted from the Swedish Cancer Register, except from comorbidity data, which was collected from the Patient administrative systems. For comorbidity analyses the Elixhauser comorbidity index was used

**Any comorbidity (any comorbidity or no comorbidity according to the Elixhauser definition)

### Survival

**Fig. 1:**
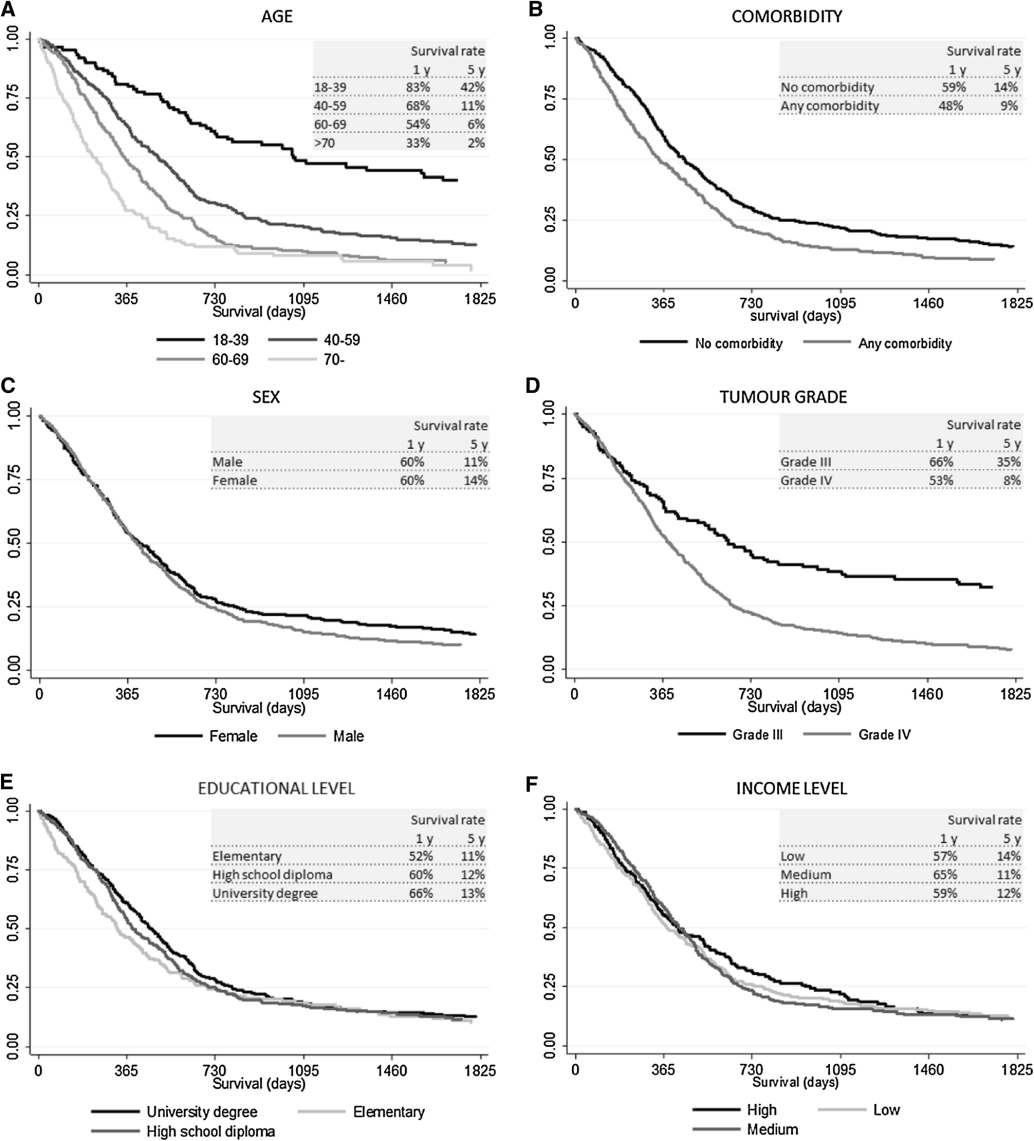
Kaplan–Meier curves for survival according to age, comorbidity status at diagnosis, sex, tumour grade, educational level or income level. Estimated survival rate at 1 year and 5 years after diagnosis is reported in each graph. Tests of statistical significance of differences between groups were performed using log-rank test for equality of survivor functions (p_(age)_ = 0.000, p_(comorbidity)_ = 0.001, p_(sex)_ = 0.121, p_(tumour grade)_ = 0.000, p_(educational level)_ = 0.095, p_(income level)_ = 0.028)

Kaplan Meier graphs of survival stratified by age, sex, comorbidity, education level, income and tumour grade are shown in Fig. 1. In univariable analysis, age, comorbidity, educational level and tumour grade was significantly associated with survival (Table 2). Patients 70 years old or older had significantly worse survival (median: 258 days) compared with younger patients (median 1105 days for age 18–39), p < 0.001. Those with low educational level had worse survival compared with patients with high educational level (median 385 days compared with 501 days), p = 0.04. Median survival for patients with comorbidity was 343 days compared with 451 days for those without comorbidity p < 0.001 and patients with high tumour grade (IV) survived in median 431 days compared with 729 days for those with tumour grade 3, p < 0.001.

**Table 2:** Cox proportional hazards model of survival (univariable and multivariable analyses) of the total study population n = 1149 in the Stockholm–Gotland region

Cox proportional hazards	Survival (univariable)		Survival (multivariable)	
	Haz. ratio (95% CI)	p value	Haz. ratio (95% CI)	p value
Age category (ref: 18–39)				
40–59	2.29 (1.83–2.87)	< **0.001**	2.15 (1.59–2.90)	< **0.001**
60–69	3.08 (2.45–3.88)	< **0.001**	3.13 (2.28–4.29)	< **0.001**
70–	4.68 (3.61–6.07)	< **0.001**	4.25 (2.99–6.02)	< **0.001**
Year of diagnosis (ref: 01/04)				
05/07	0.98 (0.81–1.19)	0.853	0.91 (0.72–1.14)	0.399
08/10	1.08 (0.91–1.28)	0.402	0.92 (0.74–1.14)	0.450
11/13	0.95 (0.80–1.15)	0.617	0.84 (0.67–1.06)	0.145
Sex				
Male	1.12 (0.99–1.27)	0.074	1.24 (1.05–1.46)	**0.010**
Comorbidity*				
Comorbid	1.33 (1.15–1.54)	< **0.001**	1.13 (0.97–1.32)	0.127
Educational level (ref: elementary)				
High school diploma	0.88 (0.74–1.05)	0.166	0.96 (0.78–1.19)	0.739
University degree	0.83 (0.70–0.99)	**0.036**	0.99 (0.79–1.24)	0.937
Income level (ref: low)				
Medium	1.04 (0.91–1.20)	0.563	0.89 (0.74–1.06)	0.191
High	0.97 (0.81–1.16)	0.749	0.76 (0.60–0.97)	**0.024**
Country of birth (ref: Sweden)				
Born in Europe (not Sweden)	1.13 (0.93–1.39)	0.219	1.23 (0.98–1.55)	0.072
Born outside Europe	0.81 (0.59–1.11)	0.192	0.94 (0.65–1.34)	0.722
Grade IV (ref: grade III)				
Grade IV	2.00 (1.58–2.53)	< **0.001**	1.57 (1.23–2.02)	< **0.001**

Bold values indicate the p value ≤ 0.05

*Any comorbidity (any comorbidity or no comorbidity according to the Elixhauser definition)

In a multivariable cox proportional hazard model of survival, older age (HR_≥70_ 4.25 (2.99–6.02) p < 0.001), male sex (HR 1.24 (1.05–1.46) p = 0.01) and high tumour grade (IV) (HR 1.57 (1.23–2.02) p < 0.001) were associated with worse survival, Table 2. High income was associated with a better survival (HR_high_ 0.76 (0.60–0.97) p = 0.02), Table 2.

### Resource use

Data on health care resource use was not available for patients living outside the region, why analysis of comorbidities and of health care resource use are based on the Stockholm population only (n = 845).

#### Outpatient visits the year before diagnosis

The multivariable analysis of outpatient visits was adjusted for age, year of diagnosis, sex, comorbidities, educational level, income level, country of birth and histopathological tumour grade, Table 3. Older patients (IRR 2.12 (1.52–2.96) p < 0.001) and patients with comorbidities (IRR 2.41 (2.05–2.84) p < 0.001) had more prediagnostic visits compared with younger patients and those without comorbidity. Older patients had in median 8 visits compared with 4 for the youngest ones. Furthermore, patients with comorbidities had 8 visits compared with 3 visits for those without comorbidity. In addition, patients diagnosed later in the study period (2011–2013) had statistically significantly fewer outpatient visits the year before HGG diagnosis compared with those diagnosed in 2001–2004 (IRR 0.74 (0.60–0.92) p = 0.007).

**Table 3:** The effect of clinical and sociodemographic factors on number of outpatient visits (univariable and multivariable regression analysis) analysed for the Stockholm population, n = 845

Negative binomial regression	Preoperative visits (univariable)		Preoperative visits (multivariable)		Postoperative visits (univariable)		Postoperative visits (multivariable)	
	IRR	p value	IRR	p value	IRR	p value	IRR	p value
Age category (ref: 18–39)								
40–59	1.53 (1.15–2.05)	**0.004**	1.40 (1.06–1.84)	**0.016**	1.08 (0.86–1.36)	0.487	1.03 (0.81–1.31)	0.814
60–69	2.31 (1.72–3.09)	< **0.001**	1.82 (1.37–2.41)	< **0.001**	1.27 (1.00-1.62)	0.052	1.17 (0.89–1.52)	0.256
70–	3.12 (2.25–4.32)	< **0.001**	2.12 (1.52–2.96)	< **0.001**	1.13 (0.80–1.58)	0.493	1.07 (0.74–1.54)	0.718
Year of diagnosis (ref: 01/04)								
05/07	1.04 (0.82–1.33)	0.748	0.93 (0.75–1.17)	0.556	1.28 (1.02–1.61)	**0.035**	1.28 (1.00-1.63)	**0.049**
08/10	0.85 (0.68–1.07)	0.165	0.77 (0.62–0.95)	**0.015**	1.25 (1.00-1.55)	0.051	1.27 (1.00-1.61)	**0.046**
11/13	0.94 (0.75–1.18)	0.584	0.74 (0.60–0.92)	**0.007**	1.46 (1.17–1.82)	**0.001**	1.53 (1.20–1.95)	**0.001**
Sex								
Male	2.67 (2.30–3.10)	< **0.001**	0.99 (0.84–1.15)	0.86	1.08 (0.92–1.27)	0.361	1.29 (1.08–1.53)	**0.004**
Comorbidity	0.80 (0.64-1.00)	**0.046**	2.41 (2.05–2.84)	< **0.001**	0.99 (0.78–1.25)	0.906	1.02 (0.86–1.20)	0.846
Educational level (ref: elementary)								
High school diploma	0.57 (0.46–0.72)	< **0.001**	0.96 (0.78–1.19)	0.708	0.99 (0.79–1.26)	0.964	1.01 (0.79–1.28)	0.964
University degree	0.76 (0.63–0.92)	**0.004**	0.83 (0.67–1.04)	0.114	1.00 (0.83–1.20)	0.968	1.06 (0.83–1.36)	0.657
Income level (ref: low)								
Medium	0.78 (0.64–0.93)	**0.007**	0.86 (0.72–1.03)	0.097	1.02 (0.85–1.23)	0.831	0.93 (0.76–1.12)	0.437
High	0.64 (0.51–0.80)	< **0.001**	0.81 (0.65–1.02)	0.071	1.03 (0.82–1.29)	0.803	0.83 (0.65–1.06)	0.136
Country of birth (ref:Sweden)								
Born in Europe (not Sweden)	1.35 (1.06–1.73)	**0.015**	1.25 (0.99–1.58)	0.065	1.28 (0.98–1.67)	0.069	1.40 (1.07–1.83)	**0.014**
Born outside Europe	1.02 (0.72–1.43)	0.93	1.08 (0.77–1.53)	0.647	0.95 (0.68–1.34)	0.77	0.93 (0.64–1.34)	0.681
Grade IV (ref: grade III)								
Grade IV	0.00 (0.00–0.00)	0.078	1.08 (0.86–1.35)	0.516	1.22 (0.99–1.51)	0.067	1.10 (0.88–1.39)	0.399

Bold values indicate the p value ≤ 0.05

The analysis of preoperative visits (1 year before diagnosis) include all 845 in the subpopulation but the postoperative visits include only the 454 patients alive after 1 year

*IRR = incidence rate ratio

**Any comorbidity (any comorbidity or no comorbidity according to the Elixhauser definition)

#### Outpatient visits the year after diagnosis

Those diagnosed the last period in the study (2011–2013) had more outpatient visits 84 versus 59 (IRR 1.53 (1.20–1.95) p < 0.001) the year after diagnosis, Table 3. In addition, male sex (76 vs 72; IRR 1.29 (1.08–1.53) p = 0.004) and being born in Europe, not Sweden (84 vs 75; IRR 1.40 (1.07–1.83) p = 0.01) was associated with a higher number of outpatient visits the year after diagnosis in multivariable analysis.

#### Inpatient care the year before diagnosis

Age, comorbidity and year of diagnosis were the only significant factors in multivariable analysis with regard to inpatient care days, Table 4. Older patients (3 vs 1, IRR_70–_ 4.79 (3.07–7.49) p < 0.001), and patients with comorbidities, (3 vs 1, IRR 1.67 (1.34–2.08) p < 0.001), had more days of inpatient care the year before diagnosis. Patients diagnosed later in the study period (2011–2013) had fewer inpatient care days the year before diagnosis (0 vs 8, IRR 0.14 (0.10–0.19) p < 0.001) than those diagnosed early (2001–2004).

**Table 4:** The effect of clinical and sociodemographic factors on number of inpatient care days (univariable and multivariable regression analysis) for the Stockholm population (n = 845)

Negative binomial regression	Preoperative days (univariable)		Preoperative days (multivariable)		Postoperative days (univariable)		Postoperative days (multivariable)	
	IRR	p value	IRR	p value	IRR	p value	IRR	p value
Age category (ref: 18–39)								
40–59	1.36 (0.90–2.05)	0.142	1.62 (1.13–2.31)	**0.009**	1.96 (1.49–2.59)	< **0.001**	2.04 (1.52–2.74)	< **0.001**
60–69	1.75 (1.16–2.66)	**0.008**	1.91 (1.31–2.79)	**0.001**	2.53 (1.89–3.40)	< **0.001**	2.40 (1.72–3.35)	< **0.001**
70–	2.57 (1.61–4.11)	< **0.001**	4.79 (3.07–7.49)	< **0.001**	1.65 (1.09–2.48)	**0.017**	1.64 (1.04–2.59)	**0.033**
Year of diagnosis (ref: 01/04)								
05/07	0.74 (0.55–1.01)	0.06	0.69 (0.51–0.92)	**0.011**	1.27 (0.95–1.69)	0.101	1.33 (0.99–1.80)	0.06
08/10	0.25 (0.19–0.34)	< **0.001**	0.18 (0.13–0.24)	< **0.001**	1.38 (1.05–1.81)	**0.022**	1.27 (0.96–1.68)	0.089
11/13	0.25 (0.19–0.34)	< **0.001**	0.14 (0.10–0.19)	< **0.001**	1.04 (0.79–1.36)	0.795	1.06 (0.80–1.42)	0.674
Sex								
Male	0.93 (0.74–1.17)	0.528	0.91 (0.74–1.13)	0.399	0.95 (0.78–1.16)	0.61	0.93 (0.76–1.14)	0.487
Comorbidity	1.72 (1.37–2.16)	< **0.001**	1.67 (1.34–2.08)	< **0.001**	1.28 (1.05–1.56)	**0.015**	1.07 (0.86–1.33)	0.528
Educational level (ref: elementary)								
High school diploma	0.71 (0.52–0.98)	**0.039**	0.78 (0.58–1.05)	0.101	1.02 (0.76–1.36)	0.919	1.04 (0.78–1.40)	0.785
University degree	0.55 (0.40–0.77)	< **0.001**	0.84 (0.62–1.15)	0.277	1.02 (0.76–1.36)	0.901	1.09 (0.80–1.47)	0.594
Income level (ref: low)								
Medium	0.84 (0.65–1.09)	0.192	0.91 (0.72–1.16)	0.451	1.16 (0.93–1.46)	0.181	1.10 (0.87–1.37)	0.433
High	0.38 (0.28–0.51)	< **0.001**	0.74 (0.54–1.02)	0.068	1.00 (0.77–1.31)	0.993	0.91 (0.68–1.22)	0.535
Country of birth (ref: Sweden)								
Born in Europe (not Sweden)	0.97 (0.68–1.38)	0.868	1.13 (0.82–1.54)	0.463	1.00 (0.73–1.38)	0.994	1.11 (0.81–1.53)	0.505
Born outside Europe	0.90 (0.55–1.46)	0.673	1.10 (0.68–1.79)	0.690	0.84 (0.56–1.27)	0.406	0.82 (0.53–1.28)	0.376
Grade IV (ref: grade III)								
Grade IV	0.94 (0.68–1.30)	0.705	1.06 (0.78–1.44)	0.709	1.41 (1.09–1.83)	**0.009**	1.21 (0.92–1.59)	0.172

Bold values indicate the p value ≤ 0.05

The analysis of preoperative days in hospital (1 year before diagnosis) include all 845 in the subpopulation but the postoperative days in hospital include only the 454 patients alive after 1 year

*IRR (CI) = incidence rate ratio with 95% confidence intervals

**Any comorbidity (any comorbidity or no comorbidity according to the Elixhauser definition)

#### Inpatient care the year after diagnosis

In multivariable analysis, age was the only significant factor associated to inpatient care the year after diagnosis. Older patients (IRR_40−59−_ (2.04 (1.52–2.74) p < 0.001) required more days of inpatient care compared with the youngest (18–39 years old), 28 versus 15 days in median, Table 4.

### Lead times

#### Lead times from diagnosis to surgery

The lead time analysis showed differences among patients depending on clinical and sociodemographic factors. Results of both univariable and multivariable analysis are reported in Table 5. The multivariable analysis of time from diagnosis (first suspicion on brain scan) to surgery showed that the oldest patients (17 vs 9 days, IRR_70−_ 1.64 (1.10–2.45) p = 0.015), patients with comorbidity (15 vs 13 days, IRR 1.53 (1.28–1.83) p = < 0.001) and patients born outside Europe (15 vs 13 days, IRR 2.79 (1.91–4.09) p = < 0.001) had to wait longer for surgery after diagnosis. The same analysis showed that patients born in Europe (not Sweden) (13 vs 14 days, IRR 0.70 (0.55–0.91) p = 0.006) and patients with grade IV tumours (13 vs 17 days, IRR 0.48 (0.36–0.63) p = < 0.001) had surgery sooner in time from first brain scan, compared with patients born in Sweden and patients with grade III tumours.

**Table 5:** The effect of clinical and sociodemographic factors on lead times in care process for the 845 patients in the Stockholm region diagnosed with high grade glioma 2001–2013

Negative binomial regression	From diagnosis to surgery (univariate)		From diagnosis to surgery (multivariate)		From surgery to start of non-surgical cancer treatment (univariate)		From surgery to start of non-surgical cancer treatment (multivariate)	
	IRR (CI)	p value	IRR (CI)	p value	IRR (CI)	p value	IRR (CI)	p value
Age category (ref: 18–39)								
40–59	0.59 (0.44–0.81)	**0.001**	1.13 (0.82–1.55)	0.455	0.42 (0.33–0.52)	**0.000**	0.67 (0.53–0.86)	**0.002**
60–69	0.55 (0.40–0.75)	**0.000**	1.30 (0.94–1.82)	0.117	0.40 (0.32–0.50)	**0.000**	0.68 (0.52–0.87)	**0.003**
70–	0.82 (0.57–1.20)	0.306	1.64 (1.10–2.45)	**0.015**	0.73 (0.52–1.02)	0.063	1.21 (0.84–1.75)	0.306
Year of diagnosis (ref: 01/04)								
05/07	1.57 (1.03–2.38)	**0.035**	0.91 (0.61–1.35)	0.635	0.43 (0.29–0.62)	**0.000**	0.64 (0.42–0.98)	**0.038**
08/10	1.40 (0.93–2.10)	0.105	1.36 (0.92–2.01)	0.127	0.26 (0.18–0.37)	**0.000**	0.42 (0.27–0.63)	**0.000**
11/13	0.91 (0.61–1.37)	0.651	0.97 (0.65–1.43)	0.870	0.29 (0.20–0.42)	**0.000**	0.40 (0.26–0.61)	**0.000**
Sex								
Male	0.83 (0.69-1.00)	**0.049**	0.91 (0.76–1.08)	0.286	1.25 (1.08–1.46)	**0.003**	1.01 (0.87–1.18)	0.863
Comorbidity*	1.57 (1.31–1.89)	**0.000**	1.53 (1.28–1.83)	**0.000**	0.81 (0.70–0.95)	**0.010**	0.94 (0.81–1.10)	0.460
Educational level (ref: elementary)								
High school diploma	1.00 (0.78–1.29)	0.997	1.11 (0.87–1.41)	0.409	1.19 (0.96–1.48)	0.114	1.06 (0.86–1.31)	0.588
University degree	0.74 (0.57–0.95)	**0.018**	1.02 (0.79–1.31)	0.879	1.32 (1.07–1.64)	**0.011**	0.98 (0.78–1.22)	0.843
Income level (ref: low)								
Medium	1.30 (1.06–1.60)	**0.010**	1.04 (0.86–1.27)	0.669	0.68 (0.57–0.82)	**0.000**	0.87 (0.73–1.04)	0.122
High	0.83 (0.66–1.05)	0.116	0.88 (0.69–1.11)	0.269	0.93 (0.77–1.13)	0.467	1.10 (0.90–1.34)	0.355
Country of birth (ref: Sweden)								
Born in Europe (not Sweden)	1.08 (0.82–1.41)	**0.000**	0.70 (0.55–0.91)	**0.006**	0.80 (0.63–1.02)	0.077	0.94 (0.76–1.16)	0.565
Born outside Europe	3.43 (2.41–4.89)	**0.000**	2.79 (1.91–4.09)	**0.000**	0.78 (0.56–1.09)	0.148	0.70 (0.50–0.97)	**0.030**
Grade IV (ref: grade III)								
Grade IV	0.37 (0.29–0.49)	**0.000**	0.48 (0.36–0.63)	**0.000**	0.50 (0.40–0.63)	0.077	0.70 (0.55–0.89)	0.003

Bold values indicate the p value ≤ 0.05

*IRR (CI) = incidence rate ratio with 95% confidence intervals

**Any comorbidity (any comorbidity or no comorbidity according to the Elixhauser definition)

#### Lead times from surgery to start of non-surgical cancer treatment

The multivariable analysis of time from surgery to start of non-surgical cancer treatment decreased over time and was significantly shorter during the last half of the study period (30 vs 60 days, IRR_11/13_ 0.40 (0.26–0.61) p = < 0.001), Table 5. This waiting time was shorter for those 60–69 years old compared with the youngest patients, 18–39 years old (34 vs 39 days, IRR_60−69_ 0.68 (0.52–0.87) p = 0.003). In addition, patients with grade IV tumours came to start non-surgical cancer treatment earlier compared with those with grade III tumours (35 vs 44 days in median, IRR 0.70 (0.55–0.89) p = 0.003) as did patients born outside of Europe compared with those born in Sweden (but similar days in median- 36 vs 35 days, IRR 0.70 (0.50–0.97) p = 0.03).

## Discussion

This is, to our knowledge, the largest population-based study addressing the impact of various clinical and sociodemographic factors on survival and resource use in patients with HGG. It is obvious, that sociodemographic status in addition to patient and disease specific factors did affect survival and health care resource use in 845 patients diagnosed with HGG during 2001–2013. A high disposable income was associated with a better survival, while older age, male sex and high tumour grade were associated with worse survival. Country of birth did not show any significant correlation with survival.

Why females and patients with a higher disposable income have a better chance of survival after HGG diagnosis can only be speculated on. However, our data is in line with those published by Sherwood et all, who also found patients with a lower income at a higher risk of earlier death [21]. One possible explanation to why high income is associated to improved survival may be a better performance status to begin with, which the observed requirement of fewer prediagnostic visits could suggest. The overall mortality and morbidity in general is well known to be higher in populations with low versus high socioeconomic status [22].

The lack of any correlation between country of birth and survival are in agreement with other studies, which found no difference in survival for patients with astrocytoma according to racial disparities [7, 23].

Field et al. published a multivariable analysis from a comprehensive dataset including 542 patients with glioblastoma. They report age, poor performance status, operation type and enrolment in clinical trial to be independent predictors for overall survival in multivariable analysis. In contrast to our data, they did not find that socioeconomic status, including income, had impact on survival [10]. However, the study populations and included variables are not exactly the same.

Our data is in line with other studies that older patients have worse survival [24]. However, this study does not include analysis of specific treatments given and it is possible that one explanation to age being highly significant is simply because older patients are more often excluded from more aggressive and possibly more effective treatments for HGG [25]. Older patients and those with comorbidities had twice as many prediagnostic visits before they came to diagnosis with HGG.

The importance of comorbidities is well known in the clinic situation, still little is known to what extent it affects outcome and resource use for HGG patients. Our previously published data, showed comorbidities to be associated with decreased survival and increased resource use in patients with primary brain tumours (not only HGG) [8]. The present analysis of resource use confirmed this in univariable analysis. However, in multivariable analysis comorbidities was not an independent prognostic factor, which may be explained by its covariation with some of the sociodemographic factors included, such as income and education.

In addition to comorbidity, other clinical and sociodemographic factors showed significant differences in relation to resource use, before and after diagnosis. The year before HGG diagnosis, patients with a higher income level as well as patients with a higher educational level had both fewer hospital admissions as well as outpatient visits. This was not significant at the multivariable level but may be interesting to further investigate in the future. Of course, the situation is complex and multifactorial but we wanted to investigate different sociodemographic factors’ possible associations with resource use and delayed diagnosis, which may have impact on survival.

Patients born in Europe, but not in Sweden, had significantly more postoperative outpatient visits. One can speculate if this may be explained by difficulties with language and communication as information, written information is usually in Swedish, or if it is due to differences in culture, but this needs to be further studied.

Our analysis of lead times in the care process was employed in order to investigate potential differences between patients with different clinical and or/sociodemographic status. Country of birth did affect time from diagnosis to surgery as well as time from surgery to start of non-surgical cancer treatment. However, the number of patients born in other countries than Sweden, especially outside Europe, is relatively limited which make it difficult to make firm conclusions. And of course, differences in a few days may not be of clinical importance and further studies are needed to see if the number and extent of differences stays the same.

Another limitation of our paper is that treatment regimen is not included in the analyses, as this stratification would render too small subgroups for robust statistical analyses. But we do know that treatment affect survival. We also lack information about other factors known to affect survival like performance status, extent of resection and molecular subtypes. On the other hand, one of the strengths is that the analysis includes all patients diagnosed with HGG between January 1st 2001 and December 31st 2013, in one large region. The unique administrative database, which covers hospital admissions as well as outpatient visits to specialists and visits in primary care, was linked to several registries by the personal identification number.

Over time we found less need for outpatient visits the year before HGG diagnosis but more outpatient visits the year after, which can be explained by improved pre diagnostic care and more interventions after diagnosis. In addition, time from surgery to start of non-surgical cancer treatment decreased over time and was significantly shorter during the last half of the study period.

The effect of sociodemographic factors on survival could potentially be reduced with increased awareness of these inequalities and relocation of resources in order to balance inequities when necessary. For example, we need to increase knowledge of when to address the health care system among all people regardless of sociodemographic status, like suggested by Whitaker et al. [4].

In conclusion, sociodemographic factors have impact on survival and resource use for patients with HGG and with increased awareness and further studies we can reduce the inequalities and further reduce clinically relevant differences.
